# Bleomycin induces senescence and repression of DNA repair via downregulation of Rad51

**DOI:** 10.1186/s10020-024-00821-y

**Published:** 2024-04-22

**Authors:** Fuqiang Chen, Wenna Zhao, Chenghong Du, Zihan Chen, Jie Du, Meijuan Zhou

**Affiliations:** 1 Department of Radiation Medicine, Guangdong Provincial Key Laboratory of Tropical Disease Research, School of Public Health, Southern Medical University, Guangzhou, 510515 Guangdong China; 2 Jiangmen Central Hospital, Affiliated Jiangmen Hospital of Sun Yat-Sen University, Jiangmen, 529030 Guangdong China

**Keywords:** Bleomycin, Lung injury, Senescence, Homologous recombination, Rad51

## Abstract

**Background:**

Bleomycin, a potent antitumor agent, is limited in clinical use due to the potential for fatal pulmonary toxicity. The accelerated DNA damage and senescence in alveolar epithelial cells (AECs) is considered a key factor in the development of lung pathology. Understanding the mechanisms for bleomycin-induced lung injury is crucial for mitigating its adverse effects.

**Methods:**

Human lung epithelial (A549) cells were exposed to bleomycin and subsequently assessed for cellular senescence, DNA damage, and double-strand break (DSB) repair. The impact of Rad51 overexpression on DSB repair and senescence in AECs was evaluated in vitro. Additionally, bleomycin was intratracheally administered in C57BL/6 mice to establish a pulmonary fibrosis model.

**Results:**

Bleomycin exposure induced dose- and time-dependent accumulation of senescence hallmarks and DNA lesions in AECs. These effects are probably due to the inhibition of Rad51 expression, consequently suppressing homologous recombination (HR) repair. Mechanistic studies revealed that bleomycin-mediated transcriptional inhibition of Rad51 might primarily result from E2F1 depletion. Furthermore, the genetic supplement of Rad51 substantially mitigated bleomycin-mediated effects on DSB repair and senescence in AECs. Notably, decreased Rad51 expression was also observed in the bleomycin‐induced mouse pulmonary fibrosis model.

**Conclusions:**

Our works suggest that the inhibition of Rad51 plays a pivotal role in bleomycin-induced AECs senescence and lung injury, offering potential strategies to alleviate the pulmonary toxicity of bleomycin.

**Supplementary Information:**

The online version contains supplementary material available at 10.1186/s10020-024-00821-y.

## Introduction

Bleomycin, a glycopeptide natural antibiotic, was originally isolated from *Streptomyces verticillus* by Umezawa in 1966 (Umezawa et al. 1966). Due to its remarkable anticancer activity, bleomycin has been extensively utilized as a chemotherapeutic agent for the treatment of various malignancies, including squamous head and neck cancers, esophageal cancers, malignant lymphomas, and choriocarcinomas (Chen and Stubbe 2005). Unfortunately, the clinical application of bleomycin is hindered by the occurrence of pulmonary toxicity with an incidence rate of up to 46%, which can even be fatal (the mortality rate is 3 to 5%) (Sleijfer 2001). A better understanding of the mechanisms underlying its toxic side effects would pave the way to optimize the therapeutic efficacy of bleomycin (Bouwman and Jonkers 2012).

Over the past decades, accumulating evidence has disclosed multiple molecular signaling pathways involved in bleomycin-induced pulmonary toxicity. It is reported to target various receptors and active cellular pathways, leading to oxidative stress, the release of inflammatory cytokines and proteases (Kim et al. 2018). However, corresponding interventions such as antioxidant or anti-inflammatory therapy remain inadequate (Oga et al. 2009; Ward et al. 1987). Cellular senescence has been recently implicated in the genesis and development of pulmonary fibrosis (Schafer et al. 2017). Increasing literature suggests that accelerated senescence of alveolar epithelial cells (AECs) serves as a potential ancestral driver in profibrotic pathogenesis (Yao et al. 2021; Lehmann et al. 2020). Senescent AECs not only lose the ability to regenerate and repair, but also promote abnormal and persistent fibroblast activation by secreting a variety of proinflammatory cytokines, pro-fibrosis factors, chemokines, and extracellular matrix remodeling proteases, known as the senescence-associated secretory phenotype (SASP) (Kadota et al. 2018; Salminen et al. 2012). Even so, the detailed mechanisms of senescence triggered by bleomycin in AECs remain poorly understood.

As a radiomimetic drug, bleomycin causes both single and double-strand breaks in the DNA molecule, with the latter thought to be the major source of cytotoxicity (Chen et al. 2008). DNA double-strand breaks (DSBs) are the most harmful type of DNA lesions, followed by chromosome instability and cellular senescence if not adequately repaired (Helbling-Leclerc et al. 2021). Eukaryotes have evolved two main pathways responsible for DSB repair: homologous recombination (HR) and non-homologous end joining (NHEJ) (Jackson and Bartek 2009). In our previous study, we developed a rapid quantitative assay for HR and NHEJ activities through CRISPR/Cas9-induced oligodeoxynucleotide (ODN)-mediated DSB repair (Du et al. 2018). Several compounds, including bleomycin, were found to notably suppress DSB repair in vitro by performing a high-throughput screening for an FDA-approved drug library (Du et al. 2021).

Herein, we confirmed the capacity of bleomycin to induce DSBs and senescence in AECs, further experiments revealed that bleomycin significantly decreased the expression of Rad51, thus suppressing HR repair in vitro. These effects seemed to be attributed to the attenuation of Rad51 protein transcription. Interestingly, restoration of Rad51 could rescue the DSB repair inhibition and cellular senescence induced by bleomycin, indicating that loss of Rad51 might contribute to bleomycin-induced lung toxicity. Our work might provide a novel insight into bleomycin-induced lung injury, which could help develop potential strategies to alleviate the pulmonary toxicity of bleomycin.

## Materials and methods

### Reagents and cell culture

Bleomycin sulfate (purity ≥ 99%) and Actinomycin D (CHD) were purchased from Selleck Chemicals (Houston, TX, USA). Cycloheximide (CHX) was obtained from Sigma (St. Louis, MO, USA). Each compound was dissolved in dimethyl sulfoxide (Sigma) and preserved at − 20 °C. Natalizumab (NTZ) and Anti-Mouse Ly-6G/Ly-6C Antibody (Anti-Ly6G) were procured from MedChemExpress (MCE, Shanghai, China). Human alveolar epithelial cell line A549 were sourced from the Cell Bank of the Chinese Academy of Sciences (Shanghai, China). HEK293T-spCas9 cells were purchased from OBiO Technology (Shanghai, China). Cells were cultured in Dulbecco's modified Eagle's medium (DMEM) supplemented with 10% fetal bovine serum (FBS), 100 mg/ml streptomycin, and 100 U/ml penicillin at 37 °C under a humidified 5% CO_2_ incubator. MLE-12 (iCell-m036) cells were acquired from iCell Bioscience (Shanghai, China) and cultured in specific MLE-12 cell medium (iCell-m036-001b).

### Senescence-associated beta-galactosidase (SA-β-gal) staining

SA-β-Gal staining was conducted following the manufacturer’s protocol (Beyotime, Shanghai, China). Initially, cell samples or frozen lung tissue sections were fixed with 4% formaldehyde for 10 min at room temperature. After fixation, the slides were rinsed with PBS and subsequently incubated with freshly prepared SA-β-Gal staining solution overnight at 37 °C. The next day, the slides were rinsed three times with PBS for 5 min each. Imaging was performed using a fluorescence microscope (Olympus, Tokyo, Japan), and the number of SA-β-Gal-positive cells was quantified in three randomly selected microscopic fields.

### 5-ethynyl-2′-deoxyuridine (EdU) assay

Cell proliferation was assessed using the BeyoClick™ EdU Cell Proliferation Kit (Beyotime). After 3 days of bleomycin treatment, cells were exposed to 10 μM EdU labeling medium at 37 °C for 2 h. Subsequently, they were immobilized, permeabilized, and stained with Click Additive Solution and Hoechst33342 solution for 30 min at room temperature in the dark. Images were captured using fluorescence microscopy (Olympus), and EdU-positive cells were counted in three randomly chosen microscopic fields.

### Cell cycle analysis

Cell cycle stages were analyzed using a propidium iodide (PI) staining protocol. After fixation in 70% ice-cold ethanol for at least 24 h, cells were stained with PI/ribonuclease staining buffer (BD Biosciences, San Jose, CA, USA) for 20 min at room temperature in the dark. The cell cycle distribution was subsequently analyzed by BD LSRFortessa X-20 flow cytometer (BD Biosciences).

### Immunofluorescence

For cell samples, cells were fixed at designated time points with 4% paraformaldehyde containing 0.2% Triton X-100 for 20 min. They were then incubated with primary antibodies against phosphorylated histone H2A variant (γH2AX), or Rad51 (all sourced from Abcam, Cambridge, UK), diluted 1:500, overnight at 4 °C. After washing, cells were further exposed to Alexa Fluor 555-conjugated goat anti-mouse IgG or Alexa Fluor 488-conjugated goat anti-rabbit IgG (both from Invitrogen, Carlsbad, CA, USA). For lung tissues, the samples were fixed in 4% paraformaldehyde and embedded in paraffin. After deparaffinating, rehydration and retrieval, tissues were incubated with primary antibodies against Podoplanin (Affinity Biosciences, Cincinnati, OH, USA) or Rad51 (Abcam), overnight at 4 °C. After washing, sections were incubated with Alexa-555- or Alexa-488-conjugated secondary antibodies (Invitrogen) for 2 h. Finally, DAPI was added onto slides for 10 min. Images were obtained with an FV3000 confocal microscope (Olympus).

### Neutral comet assay

The Comet Assay Kit (Trevigen, Gaithersburg, MD, USA) was utilized to perform neutral comet assays. A549 cells were harvested at designated time points after exposure to bleomycin. Next, 10 μl trypsinized cells were mixed with molten LMA agarose and pipetted onto Cometslides. After incubation with Lysis Solution, the slides were transferred to a horizontal electrophoresis chamber for electrophoresis at 20 V for 25 min. Finally, DNA was stained with ethidium bromide (EB, Sigma) for 20 min in darkness. Images were subsequently captured with a fluorescence microscope (Olympus) and quantitatively analyzed using CaspLab software.

### HR and NHEJ reporter plasmid assay

HR and NHEJ reporter plasmid assays were conducted as previously described (Mao et al. 2011; Li et al. 2016). To summarize, I-SceI-linearized plasmids were cotransfected with pCMV-DsRed plasmids into cells using Lipofectamine 2000 Reagent (Invitrogen) and incubated for 48 h. Fluorescently labeled cells were then analyzed using the BD LSRFFortessa X-20 Flow Cytometer (BD Biosciences). HR or NHEJ activity was determined by calculating the ratio of GFP-positive cells to DsRed-positive cells.

### Quantitative assay for HR and NHEJ activity

The measurement of HR or NHEJ activity via CRISPR/Cas9-induced dsDNA/dsODN-mediated DSB repair was performed as previously described (Du et al. 2021). In brief, sgRNA and dsDNA or dsODN were transfected into HEK293T-spCas9 cells using Lipofectamine 2000 Reagent (Invitrogen). Four hours post-transfection, the cells were treated with bleomycin for 48 h. Genomic DNA was extracted using the Genomic DNA Extraction Kit (Takara, Otsu, Japan). Finally, RT-PCR was carried out to assess the HR and NHEJ activity.

### Luciferase assay

A549 cells were seeded at a density of 5 × 10^5^ cells per well and cultured overnight. The cells were pretreated with 10 μM bleomycin 24 h prior to transfection with the luciferase reporter plasmid of Rad51-wt. The cells were collected and lysed at 48 h post-transfection, and luciferase activity was measured using the Dual-Luciferase Reporter Assay System (Promega, Madison, WI, USA) by the Tecan/Spark Luminometer (Austria). All luciferase assays were performed at least three times.

### Reverse transcription-polymerase chain reaction (RT-PCR)

Total RNA was extracted using TRIzol Reagent (Invitrogen). Reverse transcription was performed using PrimeScript RT Reagent Kit (Takara) to obtain cDNA. RT-PCR amplifications were carried out using UltraSYBR Mixture (CWBio, Beijing, China). Relative gene expression was determined by the 2^−ΔΔCt^ method and normalized with the endogenous reference gene (GAPDH). The target genes and their primer sequences are listed in Table 1.

**Table 1:** The primer sequences used for RT-PCR

Gene	Direction	Primer Sequence (5′-3′)
Human		
CXCL1	Forward	TCCTGCATCCCCCATAGTTA
	Reverse	CTTCAGGAACAGCCACCAGT
IL-1α	Forward	GGTTGAGTTTAAGCCAATCCA
	Reverse	TGCTGACCTAGGCTTGATGA
IL-1β	Forward	CTGTCCTGCGTGTTGAAAGA
	Reverse	TTGGGTAATTTTTGGGATCTACA
IL8	Forward	AGACAGCAGAGCACACAAGC
	Reverse	ATGGTTCCTTCCGGTGGT
Rad51	Forward	GGTGAAGGAAAGGCCATGTA
	Reverse	GGGTCTGGTGGTCTGTGTT
GAPDH	Forward	CATGAGAAGTATGACAACAGCCT
	Reverse	AGTCCTTCCACGATACCAAAGT
Mouse		
CXCL1	Forward	GACCATGGCTGGGATTCACC
	Reverse	CGCGACCATTCTTGAGTGTG
IL-1α	Forward	CCCATGATCTGGAAGAGACCA
	Reverse	CAAACTTCTGCCTGACGAGC
IL-1β	Forward	ATGCCACCTTTTGACAGTGATG
	Reverse	TGATGTGCTGCTGCGAGATT
IL8	Forward	CCTGATGCTCCATGGGTGAA
	Reverse	ACAGAAGCTTCATTGCCGGT
GAPDH	Forward	CAGGAGAGTGTTTCCTCGTCC
	Reverse	TGAGGTCAATGAAGGGGTCG

### Western blotting

Cells were lysed in a lysis buffer containing protease inhibitor cocktail (Beyotime). Equal amounts of total protein were separated by SDS-PAGE and electrically transferred to PVDF membranes (Millipore, Bedford, MA, USA). After blocking with 5% BSA, membranes were incubated with the following primary antibodies, all diluted to 1:1000: p21^WAF1^ (#37543), p16^ink4a^ (#18769), BRCA2 (#10741), BRCA1 (#14823), Rad50 (#3427), CtIP (#9201), NBS1 (#14956), Rad54 (#15016), Mre11 (#4895), Rad51 (#8875), RPA2 (#35869), DNA-PKcs (#4602), Ligase IV (#14649), Ku80 (#2180), Ku70 (#4588), E2F1 (#3742), γH2AX (#2577) and β-actin (#4967). These antibodies were sourced from Cell Signaling Technology (Danvers, MA, USA). After washing, goat anti-rabbit or goat anti-mouse horseradish peroxidase-conjugated secondary antibodies (Beyotime) were added for incubation at room temperature for 1 h. Immunoblotting signals were detected using an enhanced chemiluminescence method.

### Model of bleomycin-induced lung fibrosis

C57BL/6J male mice (18–20 g, 6–8 weeks) were acquired from the Laboratory Animal Centre of Southern Medical University, followed by random assignment into the control group (n = 6), the bleomycin group (n = 6), and the bleomycin + NTZ + Anti-Ly6G group (n = 6). Lung fibrosis was induced in mice by intraperitoneal injections of bleomycin (3.3 μmol/kg) dissolved in saline at day 0. On day 3, the treatments with NTZ (5 mg/kg) and Anti-Ly6G (5 μg) or vehicle were initiated. NTZ and Anti-Ly6G or the equivalent volume of vehicle were dissolved in saline immediately before the intraperitoneal injections. Mice received an injection on days 3, 6, 9, 12, 15, and 18. All mice were euthanized on day 21, and their lung tissues were collected for further analysis. All animal procedures were authorized by the Ethics Committee of Southern Medical University.

### Histological analysis and immunohistochemistry

The lung tissue samples were fixed in 4% formalin and embedded in paraffin. Histological sections (5‑µm‑thick) were stained with hematoxylin–eosin (HE) and Masson’s trichrome staining. Semiquantitative evaluation of fibrotic changes was performed according to the Ashcroft scoring criteria. Immunohistochemistry staining was performed to investigate the expression of Rad51 in vivo. In brief, lung sections were treated with 0.3% hydrogen peroxide (H_2_O_2_) for 15 min to quench the endogenous peroxidase activity. Subsequently, the sections were blocked with 5% BSA in Tris-buffered saline for 1 h and incubated with Rad51 antibodies (CST) overnight at 4 °C. After PBS wash, a peroxidase-labelled polymer conjugated to goat anti-rabbit was applied for 1 h. Then, 3,3′-diaminobenzidine tetrahydrochloride was added for 10 min at room temperature. Finally, the slides were stained with hematoxylin and visualized using a microscope (Olympus).

### Statistical analysis

Data are presented as mean ± standard deviation (SD). Statistical analysis was performed using Student’s t-test or one-way ANOVA by using SPSS 20.0 software (SPSS Inc., Chicago, IL). *p* < 0.05 were considered statistically significant.

## Results

### Bleomycin induces cellular senescence in alveolar epithelial cells

**Fig. 1:**
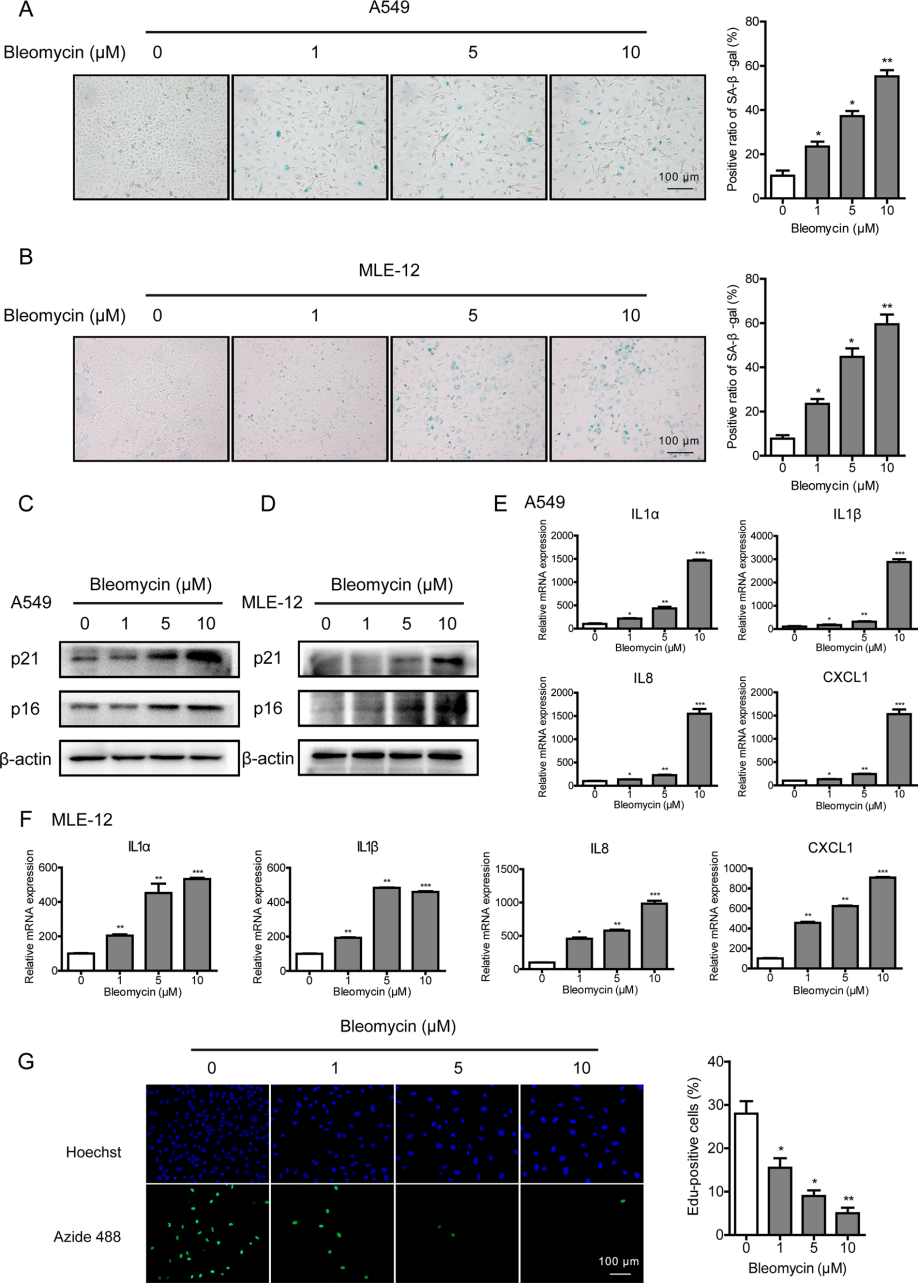
Bleomycin causes cellular senescence in AECs. **A**, **B** A549 and MLE-12 cells were treated with different concentrations of bleomycin for 3 days. SA‐β‐gal staining was performed to detect cellular senescence (magnification ×100). **C**, **D** The expression of senescence-related markers p21^WAF1^ and p16^ink4a^ in A549 and MLE-12 cells was analyzed by Western blotting. **E**, **F** The relative mRNA expression of SASP factors (IL-1α, IL-1β, IL-8, and CXCL-1) in A549 and MLE-12 cells was analyzed by RT-PCR. **G** EdU incorporation assay was performed to detect A549 cell proliferation and the EdU-positive cells were quantitated. Significance markers: *p < 0.05; **p < 0.01; ***p < 0.001 compared to control; n = 3

Bleomycin has been widely utilized to establish a cellular senescence model in experimental settings. Herein, we treated human lung epithelial A549 cells and mouse alveolar epithelial MLE-12 cells with bleomycin for 3 days at different concentrations. A549 cells were employed as a substitute for primary AECs since the response of A549 cells to bleomycin is similar with that of normal alveolar epithelial cells (Aoshiba et al. 2003; Tian et al. 2019). As expected, the intensity of positive SA‐β‐Gal staining increased in a dose- and time-dependent manner following exposure to bleomycin (Fig. 1A, B, and Additional file 1: Fig. S1A). This increase is accompanied with a distinct, flattened, and enlarged cell morphology. Meanwhile, incubation with 5 μM or 10 μM bleomycin for 72 h significantly enhanced the expression of senescence‐related markers p21^WAF1^ and p16^ink4a^ (Fig. 1C and D). Additionally, several cytokines, including IL-1α, IL-1β, IL-8, and CXCL-1, were assessed to evaluate the SASP in AECs. As depicted in Fig. 1E and F, the expression levels of all measured cytokines increased with the accumulation of bleomycin. A robust reduction in the division rate of bleomycin-treated AECs was observed by performing an EdU assay (Fig. 1G). All these findings suggested that bleomycin could effectively induce cellular senescence and trigger SASP in AECs.

### Bleomycin treatment causes persistent DNA damage

**Fig. 2:**
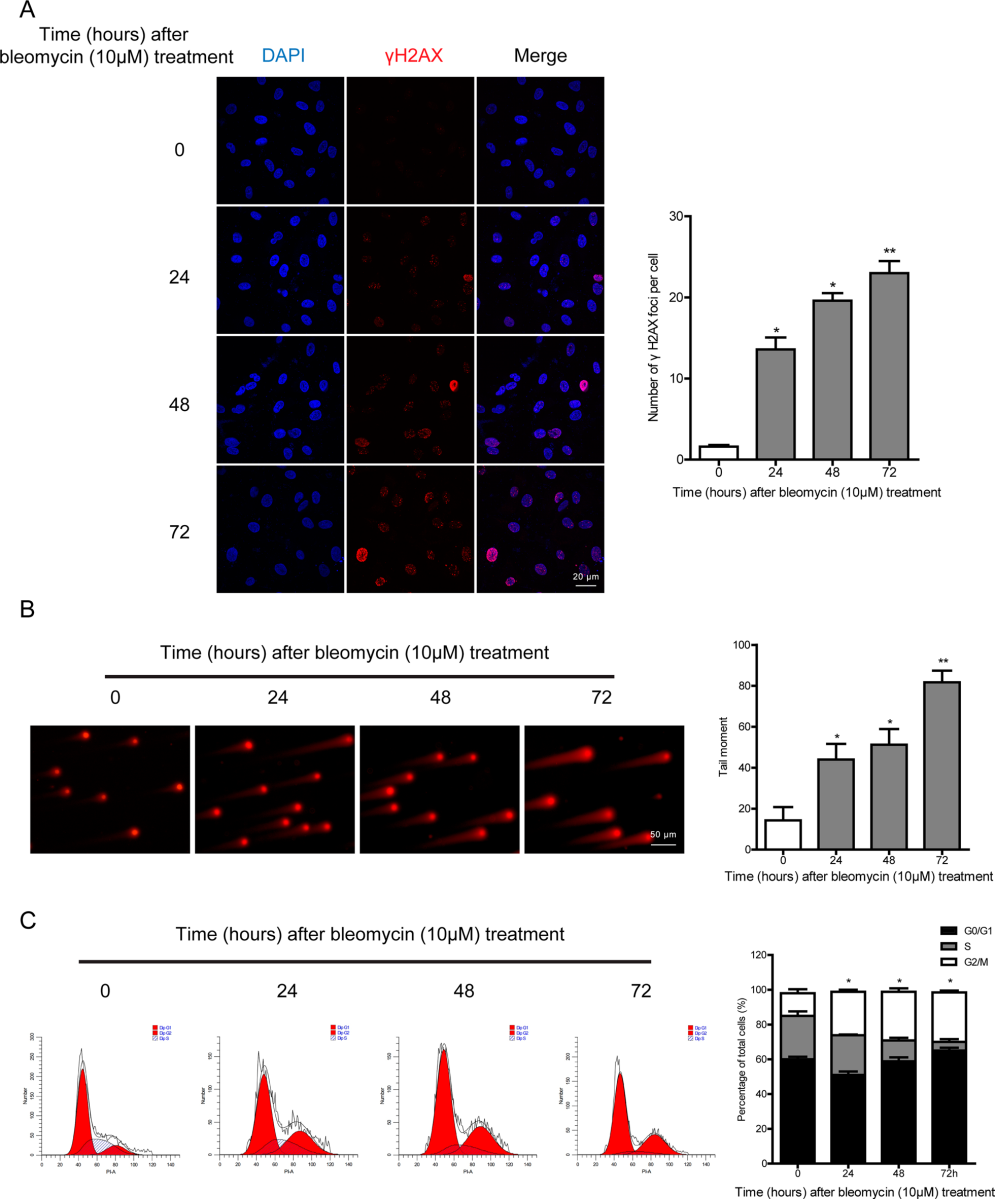
Bleomycin treatment induces sustained DNA damage. **A** A549 cells were exposed to bleomycin at a dose of 10 μM, followed by immunostaining for γH2AX foci (red) at indicated time points. **B** A549 cells were treated with bleomycin (10 μM) for the indicated time points, and then a comet assay was performed using neutral electrophoresis. **C** Cell cycle distributions were examined by flow cytometry after 1, 2, and 3 days of bleomycin exposure (10 μM). Significance markers: *p < 0.05; **p < 0.01 compared to control; n = 3

As a chemotherapeutic agent, bleomycin exerts its antitumor effects via generating DNA lesions. Since DNA damage is behind the onset of cellular senescence, we performed immunofluorescence to detect the formation of γH2AX foci. Bleomycin-treated cells showed higher numbers of foci at 24 h compared with that of the vehicle-treated group (Fig. 2A). Furthermore, we found significant higher levels of DNA damage in presence of continuous exposure to bleomycin (Fig. 2A). Similarly, neutral comet assays confirmed the persistent DNA damage caused by bleomycin, as evidenced by the elevated DNA content in the comet tails (Fig. 2B). To evaluate the effect of bleomycin on cell cycle progression, A549 cells were harvested at 1, 2, and 3 days after bleomycin treatment and subsequently PI stained. As shown by flow cytometric analysis (Fig. 2C), cells in G2/M phase accumulated in the bleomycin-treated group. Taken together, these results provided additional support that continuous bleomycin treatment could lead to persistent DNA damage, along with increased secretion of SASP-associated cytokines mentioned above.

### Bleomycin impairs DSB repair efficiency by HR not NHEJ

**Fig. 3:**
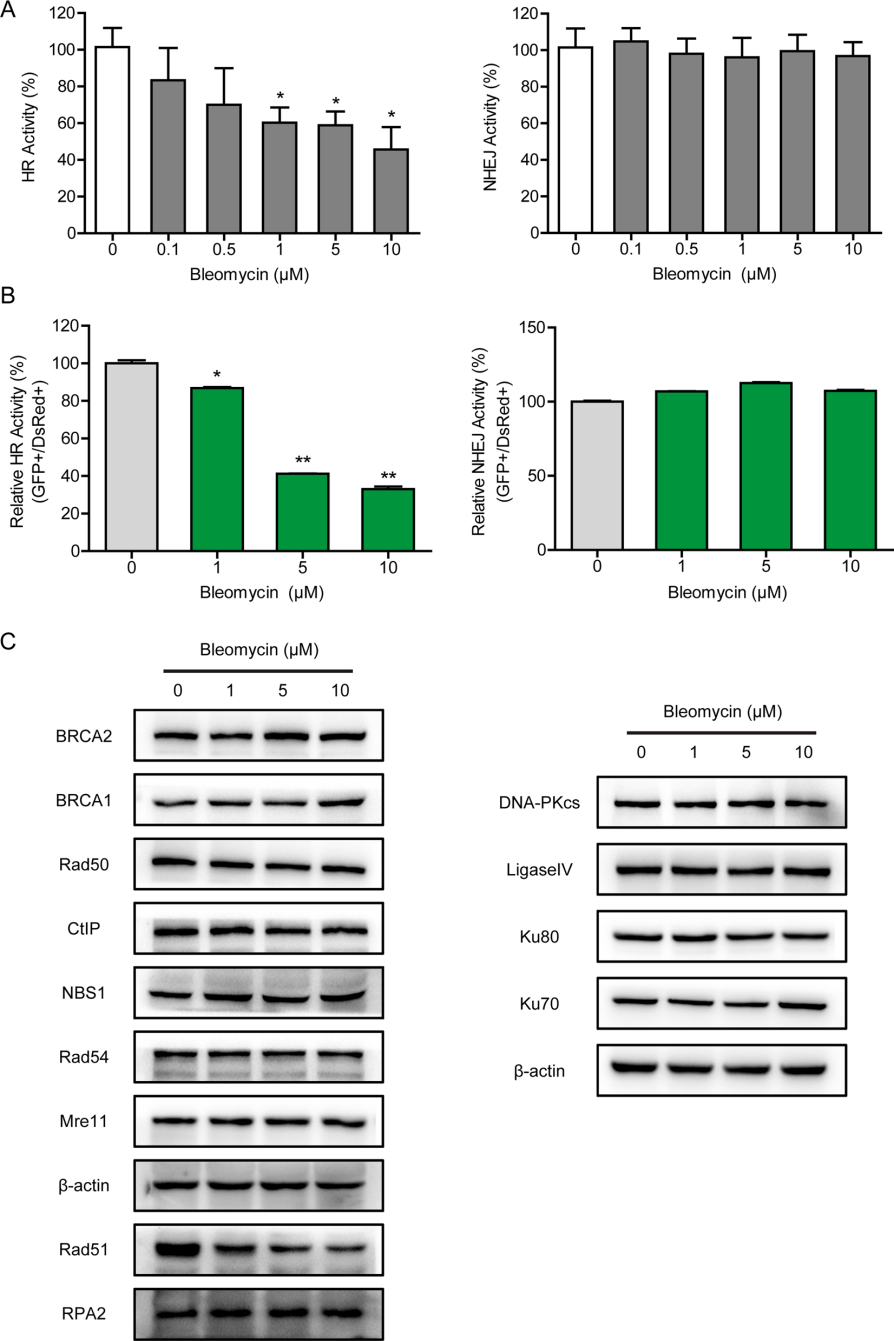
Bleomycin impairs DSB repair efficiency by HR. **A** HEK293T-spCas9 cells were co-transfected with sgRNA and dsDNA/dsODN, followed by treatment of bleomycin for 48 h, and genomic DNA was extracted for RT-PCR analysis. The HR-suppressive effects of bleomycin were shown in a dose-dependent manner. **B** Measurement of HR and NHEJ activities by using the GFP-based HR/NHEJ reporter assay. HEK293T-spCas9 cells were transfected with I-SceI-linearized plasmids and incubated with bleomycin at different concentrations for 48 h. A DsRed-expressing plasmid was co-transfected as transfection control. The ratio of GFP-positive cells to DsRed-positive cells was measured by flow cytometry. **C** A549 cells were treated with the indicated concentrations of bleomycin for 24 h, the expression levels of important HR (left) and NHEJ (right) pathway-associated factors were analyzed by Western blotting. Significance markers: *p < 0.05; **p < 0.01 compared to control; n = 3

Recent evidence has highlighted DSB as the critical lesion among the cytotoxic effects of bleomycin. To further examine the potential impact of bleomycin on HR and/or NHEJ, we employed our well-established detecting system to measure the efficiency of HR and NHEJ repair (Du et al. 2021). As illustrated in Fig. 3A, bleomycin exhibited concentration-dependent suppressive effects on HR activity while having no impact on NHEJ. This investigation was further corroborated by using the HR and NHEJ reporter assay, in which successful HR/NHEJ repair of I-SceI nuclease-induced DSBs would restore the expression of the intact GFP gene (Fig. 3B). To elucidate the mechanism of bleomycin on DSB repair, we initially examined the expression levels of important proteins involved in HR and NHEJ repair. Western blotting indicated that bleomycin treatment did not affect the expression of HR factors (BRCA2, BRCA1, Rad50, CtIP, NBS1, Rad54, Mre11, and RPA2) or NHEJ factors (DNA-PKcs, Ligase IV, Ku80, and Ku70) (Fig. 3C). Surprisingly, Rad51, a key protein in HR repair, was significantly lowered by bleomycin treatment (Fig. 3C).

### Bleomycin affects DNA repair by downregulating the expression of Rad51 at the transcriptional level

**Fig. 4:**
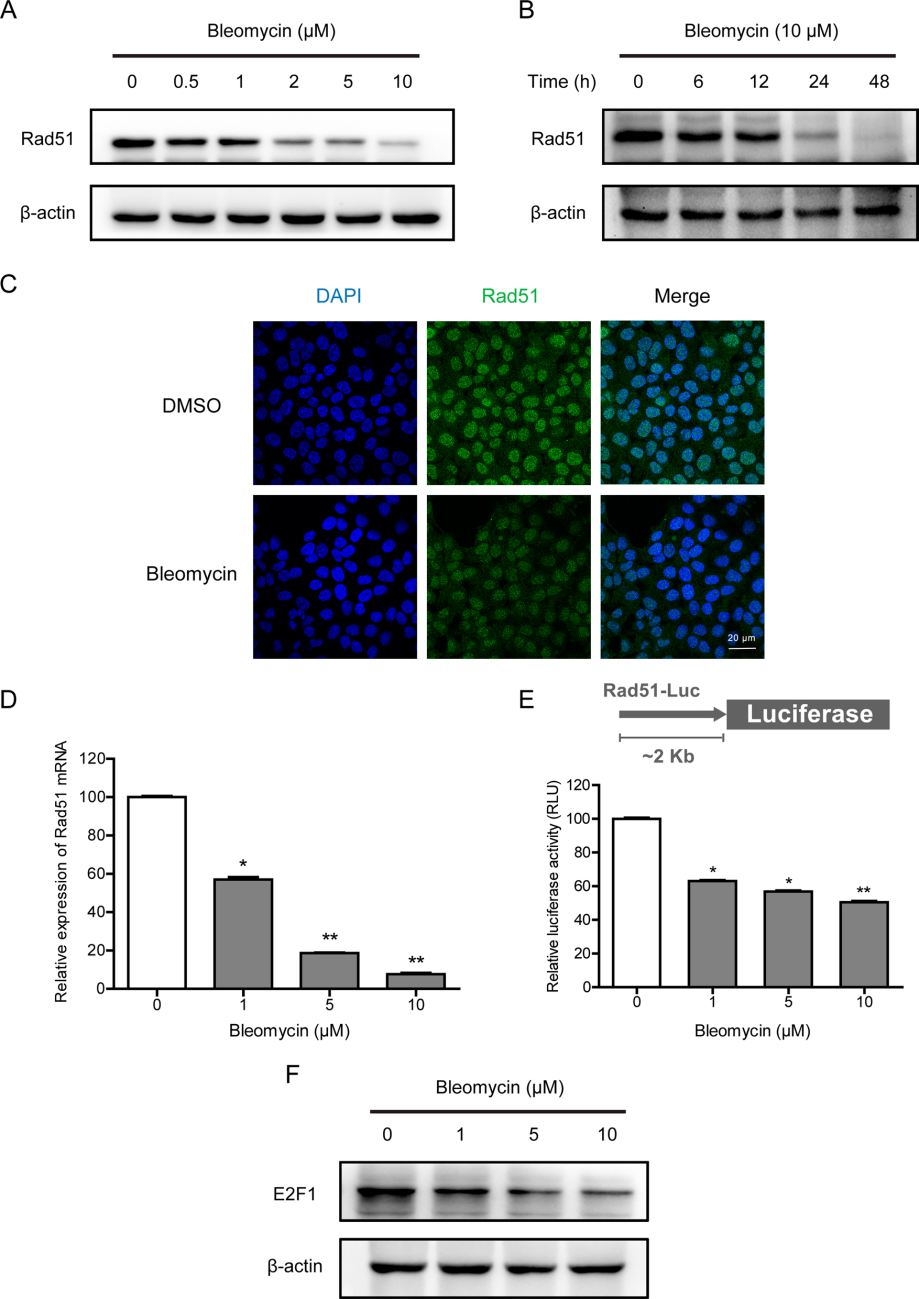
Bleomycin impacts DNA repair by transcriptionally downregulating the expression of Rad51. **A**, **B** A549 cells were treated with bleomycin at the indicated concentrations and time, the expression of Rad51 was determined by Western blotting. **C** A549 cells treated with vehicle or 10 μM bleomycin for 24 h were analyzed by immunofluorescence using anti-Rad51 antibodies. **D** A549 cells were exposed to the indicated doses of bleomycin for 24 h, and Rad51 mRNA was assessed by RT-PCR. **E** A549 cells were treated with 10 μM bleomycin 24 h before transfection with the luciferase reporter. Relative luciferase activity was determined by normalizing firefly luciferase against renilla luciferase activity. **F** The expression levels of E2F1 after 24 h of bleomycin exposure were analyzed by Western blotting. Significance markers: *p < 0.05; **p < 0.01 compared to control; n = 3

Since Rad51 serves as a critical role in the maintenance of genome stability, we next examined the regulatory effects of bleomycin on Rad51 in AECs. Bleomycin treatment noteworthily decreased Rad51 expression in a dose-dependent manner (Fig. 4A). Time-course examination revealed that suppression of Rad51 could occur as early as 6 h after exposure to bleomycin (Fig. 4B). Immunofluorescent staining confirmed the reduced Rad51 levels in AECs treated with 10 μM bleomycin for 24 h (Fig. 4C). To elucidate the mechanism by which bleomycin decreased Rad51 expression, we first performed RT-PCR to determine whether bleomycin has a direct effect on the mRNA level of Rad51. As depicted in Fig. 4D, the downregulation of Rad51 protein levels by bleomycin was the result of a decrease at the transcriptional level. No surprise, it seemed irrelevant to the degradation rates of protein or mRNA (Additional file 1: Fig. S2A and B). Additionally, luciferase assays demonstrated that bleomycin impaired Rad51 promoter activity (Fig. 4E). Considering that ChIP-chip experimentation has previously characterized E2F1 as a predominant transcription factor for Rad51 (Ren et al. 2002), we further measured the expression of E2F1 (Fig. 4F). Collectively, treatment with bleomycin was capable to block the transcription of Rad51 through dose-dependent repression of E2F1.

### Rad51 overexpression abolishes bleomycin-mediated effects on DNA repair and senescence

**Fig. 5:**
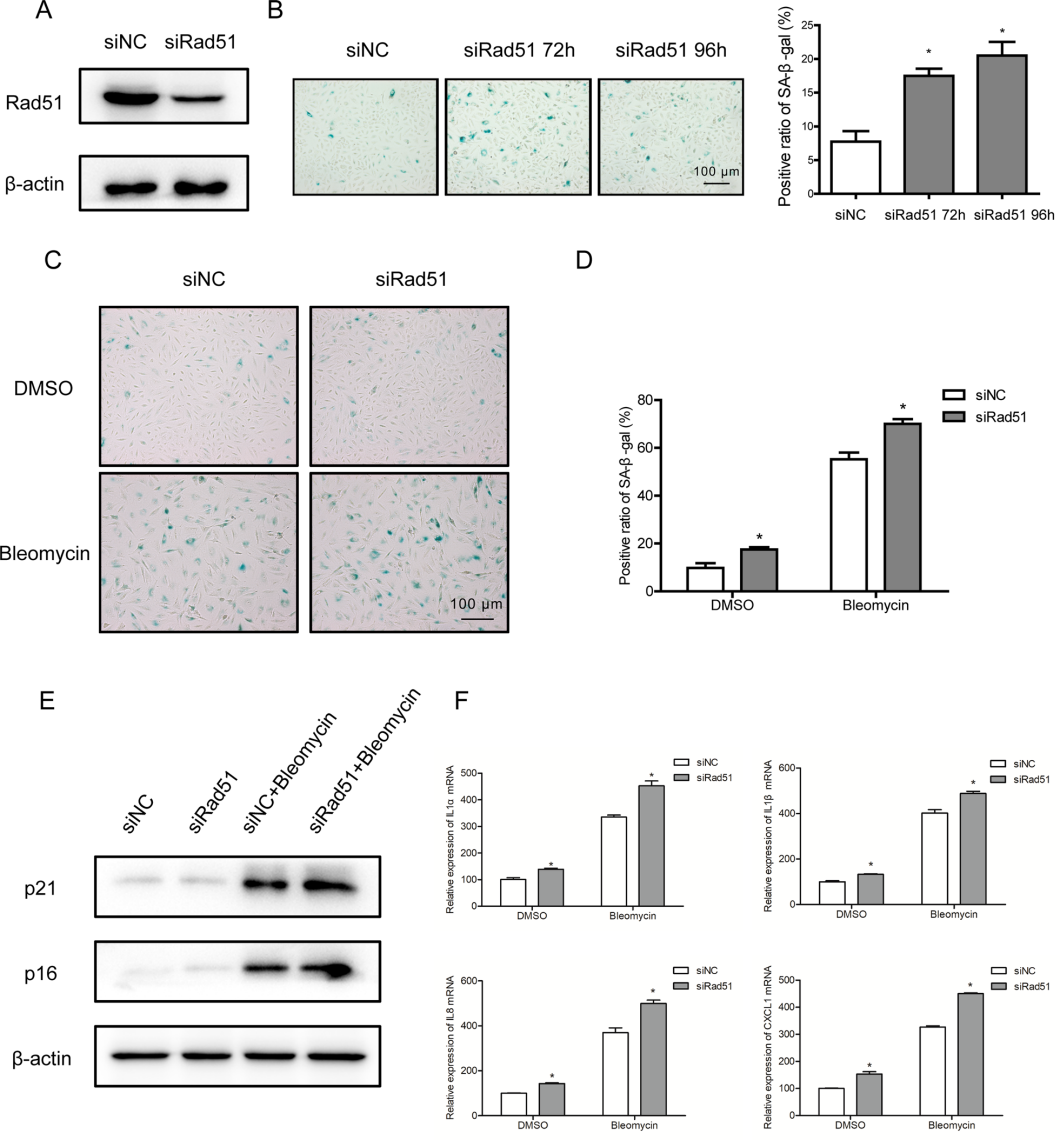
Silencing of Rad51 enhances bleomycin-induced cellular senescence in AECs. **A** Western blotting of the Rad51 expression in A549 cells transfected with control or Rad51 siRNA. **B** A549 cells were transfected with control or Rad51 siRNA for the indicated time, followed by SA-β-gal staining (magnification ×100). **C**, **D** After transfection with control or Rad51 siRNA, A549 cells were incubated with bleomycin for 72 h. SA‐β‐gal staining was performed to detect cell senescence (magnification ×100). **E** Western blotting of p21^WAF1^ and p16^ink4a^ expression in bleomycin-treated A549 cells with control or Rad51 siRNA transfected. **F** RT-PCR analysis of SASP factors (IL-1α, IL-1β, IL-8, and CXCL-1) in bleomycin-treated A549 cells with control or Rad51 siRNA transfected. Significance markers: *p < 0.05 compared to control; n = 3

Currently, it was proposed that specifically reducing Rad51-mediated HR could lead to premature aging and inflammation in growing mice (Matos-Rodrigues et al. 2023). To determine whether the down-regulation of Rad51 could induce senescence in vitro, Rad51 gene expression knockdown was performed in A549. The intensity of positive SA‐β‐Gal staining increased after Rad51 knockdown, accompanied with the elevated SASP factors (Fig. 5A, B, and Additional file 1: Fig. S2C). Additionally, we found that Rad51 silencing could accelerate AECs senescence induced by bleomycin, as illustrated by SA-β-Gal staining and the expression levels of aging‐related markers (Fig. 5C–F).

**Fig. 6:**
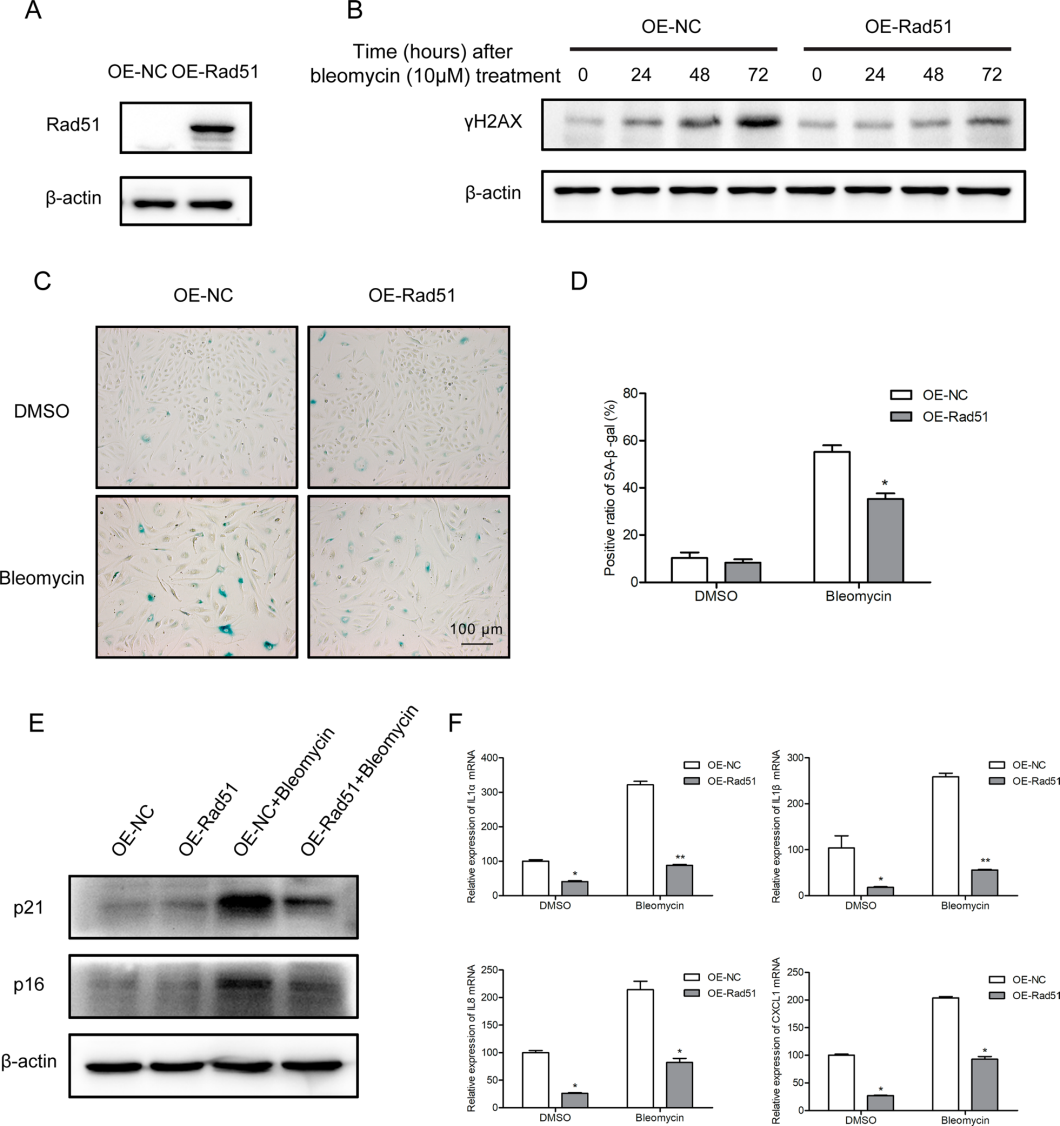
Overexpression of Rad51 eliminates bleomycin-induced inhibition of DNA repair and senescence in AECs. **A** Western blotting of the Rad51 expression in A549 cells infected with Rad51 expression vector. **B** Cells were transfected with control vector or Rad51 expression vector, and further treated with bleomycin (10 μM) for 24, 48, or 72 h. The expression of γH2AX was measured by Western blotting. **C**, **D** After transfection with control vector or Rad51 expression vector, A549 cells were incubated with bleomycin for 72 h. SA‐β‐gal staining was performed to detect cell senescence (magnification ×100). **E** Western blotting of p21^WAF1^ and p16^ink4a^ expression in bleomycin-treated A549 cells with control or Rad51 vectors transfected. **F** RT-PCR analysis of SASP factors (IL-1α, IL-1β, IL-8, and CXCL-1) in bleomycin-treated A549 cells with control or Rad51 vectors transfected. Significance markers: *p < 0.05; **p < 0.01 compared to control; n = 3

Given that Rad51 showed robust decline following bleomycin treatment, we therefore hypothesized that restoration of Rad51 may mitigate the cellular senescence induced by bleomycin. To test this hypothesis, we constructed a vector encoding Rad51 driven by the CMV promoter and first examined the impact of over-expressed Rad51 on DNA repair efficiency following exposure to bleomycin (Fig. 6A). Forty-eight hours after transfection, we treated AECs with bleomycin, followed by the analysis of γH2AX expression at indicated time points. As expected, overexpression of Rad51 significantly promoted DSB repair efficiency in bleomycin-treated AECs (Fig. 6B). Subsequently, we investigated whether restoration of Rad51 could potentially ameliorate the onset of bleomycin-mediated cell senescence. As depicted in Fig. 6C and D, the intensity of positive SA-β-Gal staining decreased when Rad51 was overexpressed. Similarly, we observed remarkable reductions in senescent markers p16^ink4a^ and p21^WAF1^, as well as SASP-associated markers after the genetic manipulation of Rad51 (Fig. 6E and F). Additionally, EdU incorporation assay showed that supplementation with Rad51 significantly stimulated the division rate compared to the control group in the presence of bleomycin (Additional file 1: Fig. S2D). These results support our hypothesis that augmenting DSB repair through reinforcing Rad51 might be a promising approach to alleviate bleomycin-induced cellular senescence.

### Rad51 is decreased in the bleomycin-induced mouse pulmonary fibrosis model

**Fig. 7:**
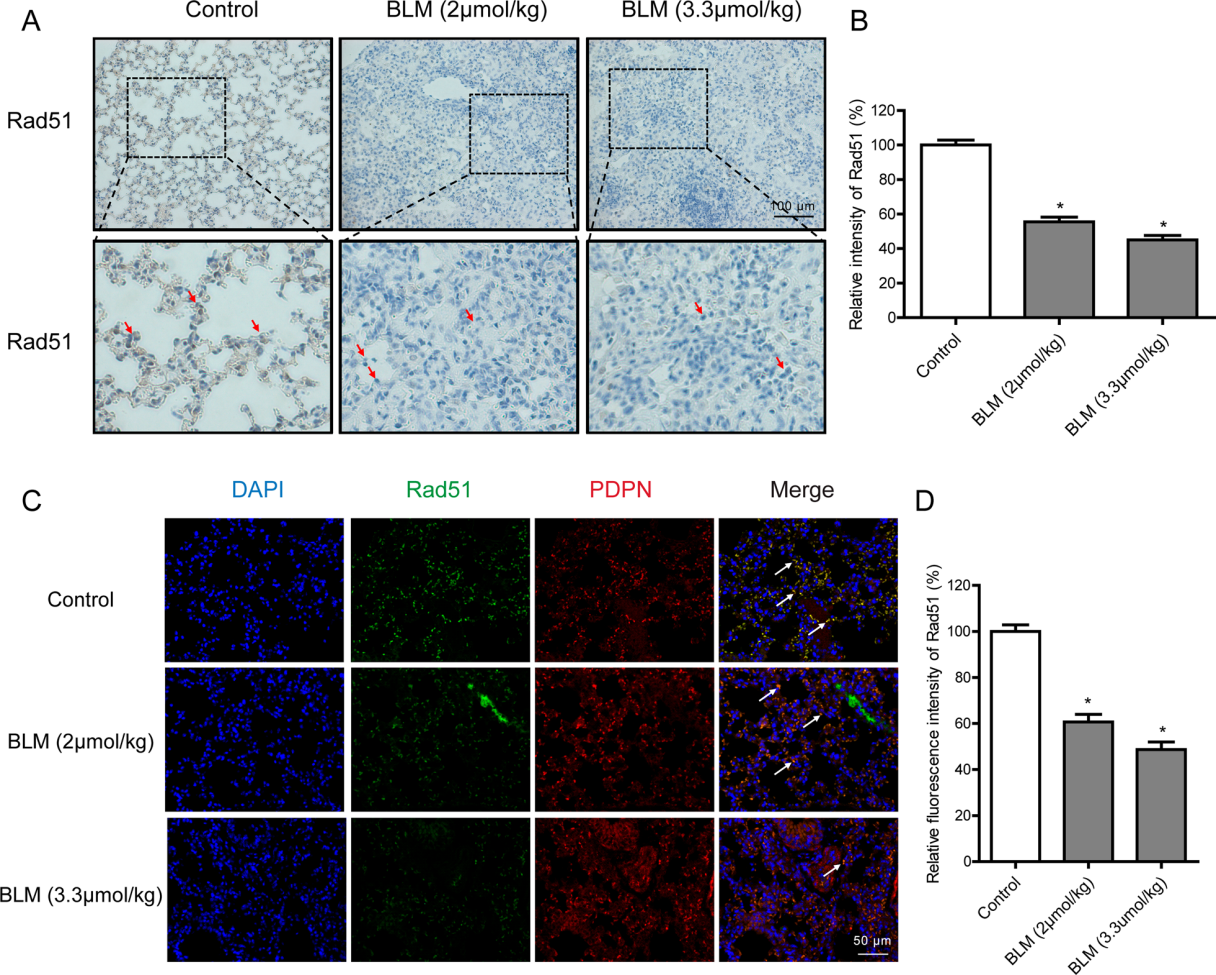
Reduction of Rad51 expression in the bleomycin-induced mouse pulmonary fibrosis model. **A** Representative immunohistochemical analysis of Rad51 (brown) in lung sections. The control group showed strong positive staining for Rad51 compared to the bleomycin group (Arrows). **B** The semi-quantitative assessment of immunohistochemistry was determined by ImageJ software. **C** Representative images of Rad51 expression in AECs by co-immunofluorescence staining with both Rad51 (green) and PDPN (red). **D** The intensity of Rad51 immunofluorescence was quantified by ImageJ software. Significance markers: *p < 0.05 compared to control; n = 6

Bleomycin-induced mouse lung injury model has been widely utilized in the research of pulmonary fibrosis. Herein, we also established a bleomycin-induced pulmonary fibrosis model in C57BL/6 mice through intratracheal instillation. After exposure to bleomycin for 21 days, we euthanized the mice and isolated their lung tissue for analysis. As shown in Additional file 1: Fig. S1B, HE and Masson staining revealed the destruction of alveolar structure and the appearance of blue-stained collagen fibers. And SA‐β‐Gal staining showed increased amounts of blue-stained senescent cells compared with the saline control. Importantly, the decreased expression of Rad51 was also encountered in the bleomycin group, which was dyed brown (Fig. 7A and B). With double-label immunofluorescent staining method, to assess the presence of Rad51 in AECs, the expression levels of podoplanin (a specific marker of AECs) and Rad51 were further analyzed in lung tissues (Fig. 7C and D).

**Fig. 8:**
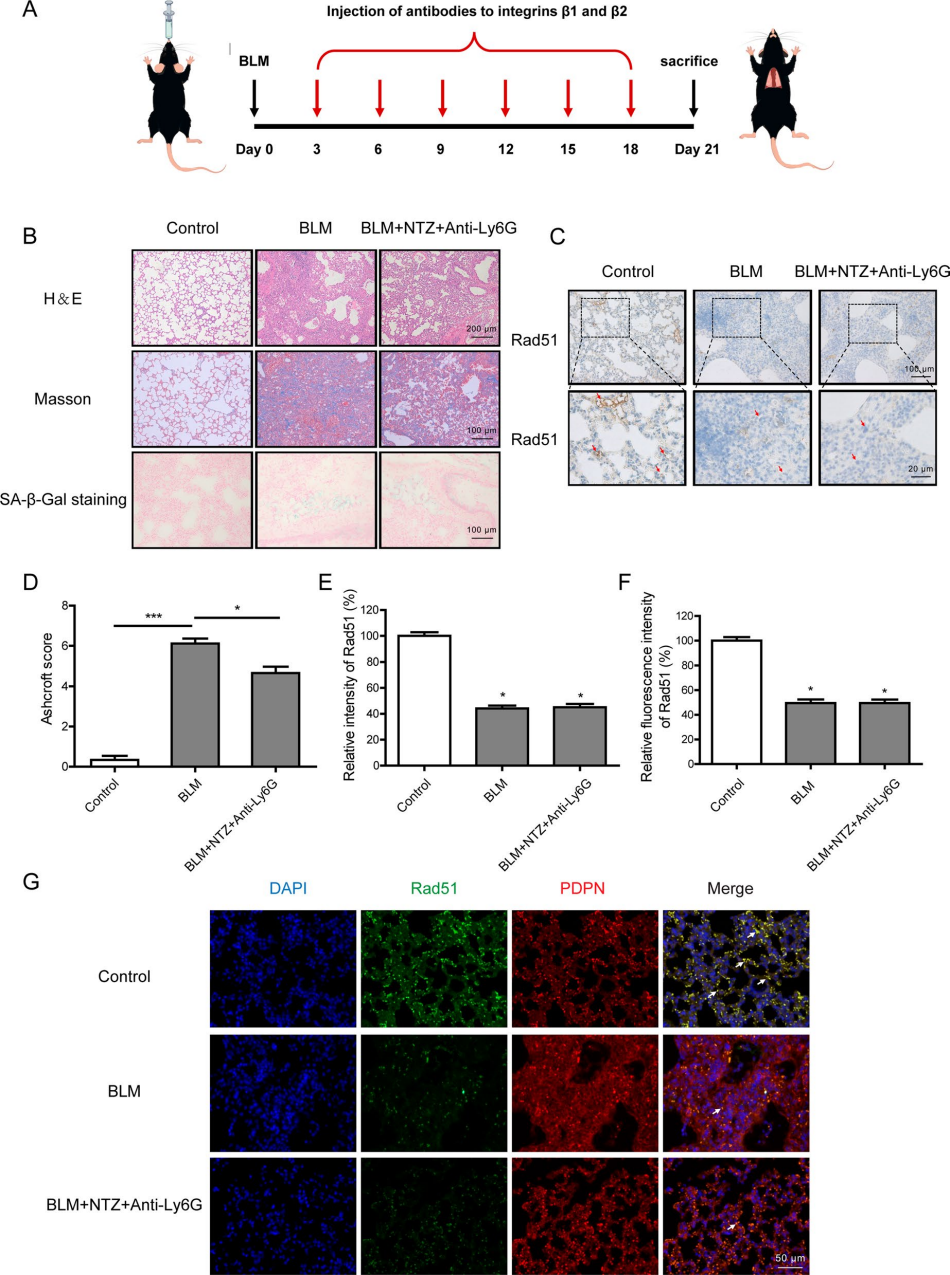
Increased senescence markers and reduced Rad51 Expression in vivo. **A** Schematic picture of the animal experiment. **B** Representative HE (Upper panel), Masson staining (Middle panel), and SA-β-Gal staining (Lower panel) of harvested mouse lung tissues. **C** Representative immunohistochemical analysis of Rad51 (brown) in lung sections. The control group showed strong positive staining for Rad51 compared to the BLM and BLM + NTZ + Anti-Ly6G group (Arrows). **D** Quantitative analysis of fibrosis was performed using the Ashcroft score. **E** The semi-quantitative assessment of immunohistochemistry was determined by ImageJ software. **F** The intensity of Rad51 immunofluorescence was quantified by ImageJ software. **G** Representative images of Rad51 expression in AECs by co-immunofluorescence staining with both Rad51 (green) and PDPN (red). Significance markers: *p < 0.05; ***p < 0.001 compared to control; n = 6

Inflammatory leukocytes such as neutrophils, eosinophils, and monocytes have been implicated as mediators of bleomycin-induced lung injury (Wang et al. 2011). In order to clarify that the reduction of Rad51 expression in mice is due to the direct effect of bleomycin on AECs, we further check Rad51 expression after injection of antibodies to integrin β1 and β2. Natalizumab (NTZ) and Anti-Mouse Ly-6G/Ly-6C Antibody (Anti-Ly6G) were used to block monocyte recruitment and neutrophil recruitment, respectively (Fig. 8A). As shown in Fig. 8B and D, to some extent, anti-inflammatory treatment could ameliorate the pulmonary toxicity caused by bleomycin. However, the decreasing Rad51 expression induced by bleomycin was unchanged following exposure to NTZ and Anti-Ly6G, indicating that bleomycin could directly suppress the expression of Rad51 (Fig. 8C and E). Meanwhile, the results were further confirmed by immunofluorescence staining (Fig. 8F and G).

## Discussion

Despite being an effective anti-tumor chemotherapeutic drug, bleomycin is feared for its induction of sometimes fatal pulmonary toxicity. Hence, understanding the molecular mechanism of bleomycin to induce lung injury will contribute to its therapeutic application and related disease drug discovery. In the current study, we demonstrated that bleomycin was capable of reducing the expression of Rad51, at the transcriptional level, thereby blocking the HR repair pathway in AECs. Rad51 overexpression abolished bleomycin-mediated effects on DNA repair and cellular senescence, implying that loss of Rad51 might be involved in bleomycin-induced pathological process. Further examination revealed decreased Rad51 expression in the pulmonary fibrosis mouse model induced by intratracheal injection of bleomycin.

Idiopathic pulmonary fibrosis (IPF) is an irreversible and fatal interstitial lung disease that shows a pathological process similar to that of bleomycin-mediated lung toxicity. Up-regulation of senescence markers such as p16^ink4a^ and p21^WAF1^ has been disclosed in the lung epithelium of IPF patients together with bleomycin‐induced mouse pulmonary fibrosis models (Tian et al. 2019; Kuwano et al. 1996; Yanai et al. 2015). Consistent with the previous studies (Aoshiba et al. 2003), our finding confirmed that continuous exposure to bleomycin displayed senescence hallmarks in alveolar epithelial cells in vitro and in vivo (Fig. 1 and Additional file 1: Fig. S1). Multiple mechanisms can contribute to the induction of senescence, of which DNA damage is the most likely initiating trigger of ageing (Munoz-Espin and Serrano 2014). In fact, persistent activation of DNA damage response (DDR) is widely considered as a common feature of cellular senescence, while increased amounts of γH2AX have been detected in epithelial cells of IPF patients co-localizing with p16^ink4a^ staining (Lehmann et al. 2017). In line with this, our experimental evidence exhibited time-dependent accumulating DNA lesions in the presence of bleomycin, which would further confirm the contact between DNA damage and ageing (Fig. 2).

The critical lesions among the cytotoxic effects of bleomycin are DNA double-strand breaks, which could lead to severe consequences such as SIPS if not be appropriately repaired. In previous studies, we established a cell-based quantitative assay for HR and NHEJ and performed a high-throughput screening for an FDA-approved drug library. Omipalisib was identified as a potent radio- and chemosensitizer, based on its ability to reduce NHEJ activity via DNA-PKcs inhibition (Du et al. 2018; Du et al. 2021). Unexpectedly, bleomycin, a clastogenic compound, showed remarkable inhibitory effects on HR repair at the working concentration. In addition to its known actions, bleomycin was found to suppress HR activity in a dose-dependent manner (Fig. 3A and B) but have no significant effect on NHEJ. Mechanistic studies indicated that bleomycin treatment did not display an obvious effect on the expression of HR and NHEJ pathway-associated factors except Rad51. The Rad51 protein level was reduced by bleomycin in vitro and in vivo, on account of the decrease at the transcriptional level (Figs. 4 and 7). And deletion of Rad51 could mildly drive AECs senescence and augment bleomycin-induced SASP (Fig. 5). Further CRISPR/Cas9-mediated genetic manipulation of Rad51 should be performed efficiently in the future. Moreover, human transcription factor E2F1 has been assumed to be involved in the regulation of HR activity and Rad51 foci formation. The expression of HR factors including Rad51 was downregulated in the absence of E2F1 (Choi and Kim 2019). Specifically, we observed a decline in E2F1 protein expression following exposure to bleomycin, suggesting that bleomycin-mediated transcriptional inhibition of Rad51 might be partly attributable to the depletion of E2F1. Nonetheless, the detailed molecular mechanisms of bleomycin-mediated Rad51 suppression need to be further studied.

The biological mechanisms underlying bleomycin-induced pulmonary injury have not been entrenched so far. Inflammation serves as a major component that is orchestrated in part by activated leukocytes, which are considered to contribute to the fibrotic processes (Oury et al. 2001). Then we examined Rad51 expression after blocking leukocyte recruitment to verify the direct inhibition of bleomycin on Rad51 in the mouse model. Even though anti-inflammatory agents such as NTZ and Anti-Ly6G could partially alleviate bleomycin-induced pulmonary injury (Pyka-Fosciak et al. 2020; Wang et al. 2012), the reduction of Rad51 was not affected (Fig. 8). Currently, it was suggested that the stalling of DNA repair process triggers senescence-associated inflammatory cytokine secretion and aggravates the lung pathology induced by bleomycin (Rodier et al. 2009; Guijarro et al. 2018). In the case of that, activated DNA repair might hold great potential to delay ageing and age-associated pathologies (Schumacher et al. 2021). For instance, lycorine hydrochloride improves the DSB repair efficiency via promoting the expression of SIRT1 and SIRT6, thereby restraining radiation-induced senescence (Zhang et al. 2021). Consistently, our results demonstrated that supplementing with Rad51 rescued stalled DSB repair in bleomycin-treated AECs, accompanied by the diminution of senescence biomarkers and SASP cytokines (Fig. 6). As a matter of course, further rescue experiments in vivo should be carried out to confirm if targeting Rad51 could ameliorate cellular senescence caused by bleomycin.

In summary, despite the widespread use of bleomycin for inducing lung fibrosis in experimental models, the underlying mechanisms of bleomycin-mediated lung toxicity remain unsettled. The current study demonstrated that bleomycin blocks DSB repair by HR not NHEJ via directly suppressing Rad51 expression at the transcriptional level, resulting in AECs senescence and the associated SASP. Our work supports a vital role for Rad51 in modulating bleomycin-induced lung injury, which may assist in opening new perspectives for the development of potential strategies to minimize pulmonary toxicity of bleomycin.

### Supplementary Information

**Additional file 1: Figure S1.** Analysis of bleomycin-induced cellular senescence and pulmonary fibrosis. **A** A549 cells were incubated with bleomycin at the indicated time (0, 24, 48, and 72 h), followed by SA-β-gal staining (magnification ×100). **B** Representative HE (Upper panel), Masson staining (Middle panel), and SA-β-Gal staining (Lower panel) of harvested mouse lung tissues. Fibrosis was evaluated using the Ashcroft score. Significance markers: *p < 0.05; **p < 0.01; ***p < 0.001 compared to control; n = 6. **Figure S2.** Rad51 protein and RNA stability assay, RT-PCR analysis, and EdU incorporation assay. **A** A549 cells were treated with 100 μg/ml CHX alone or in combination with bleomycin for 0, 3, 6, and 12 h. Then Rad51 protein expression levels were assessed by Western blotting. **B** A549 cells were treated with 5 μg/ml CHD alone or in combination with bleomycin for 0, 1, 3, 6, and 12 hours. The relative mRNA expression of Rad51 was assessed by RT-PCR. **C** RT-PCR analysis of SASP factors (IL-1α, IL-1β, IL-8, and CXCL-1) in A549 cells transfected with control or Rad51 siRNA at the indicated time. **D** Analysis of cell division on bleomycin-treated A549 cells with control or Rad51 vectors transfected by performing the EdU incorporation assay. Significance markers: *p < 0.05 compared to control; n = 3.

## Data Availability

All data generated or analysed during this study are included in this published article [and its additional files].
