# Supplementary figures and images for: Bleomycin induces senescence and repression of DNA repair via downregulation of Rad51
Source: Mol Med. 2024 Apr 22;30:54. doi: 10.1186/s10020-024-00821-y (PMC11036784; doi:10.1186/s10020-024-00821-y)

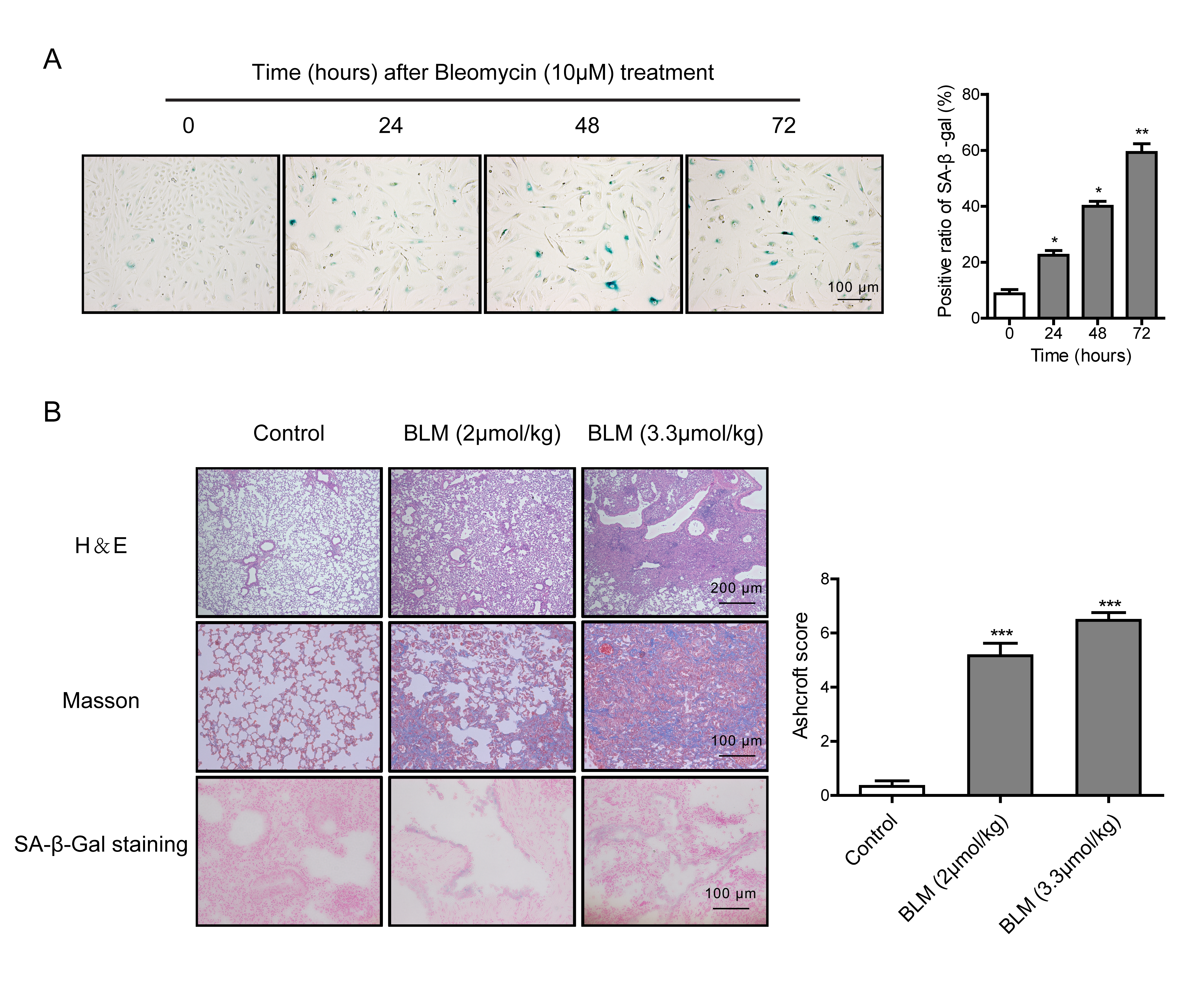

Supplement: Supplementary file 1 — Additional file 1: Figure S1. Analysis of bleomycin-induced cellular senescence and pulmonary fibrosis. A A549 cells were incubated with bleomycin at the indicated time (0, 24, 48, and 72 h), followed by SA-β-gal staining (magnification ×100). B Representative HE (Upper panel), Masson staining (Middle panel), and SA-β-Gal staining (Lower panel) of harvested mouse lung tissues. Fibrosis was evaluated using the Ashcroft score. Significance markers: *p < 0.05; **p < 0.01; ***p < 0.001 compared to control; n = 6. Figure S2. Rad51 protein and RNA stability assay, RT-PCR analysis, and EdU incorporation assay. A A549 cells were treated with 100 μg/ml CHX alone or in combination with bleomycin for 0, 3, 6, and 12 h. Then Rad51 protein expression levels were assessed by Western blotting. B A549 cells were treated with 5 μg/ml CHD alone or in combination with bleomycin for 0, 1, 3, 6, and 12 hours. The relative mRNA expression of Rad51 was assessed by RT-PCR. C RT-PCR analysis of SASP factors (IL-1α, IL-1β, IL-8, and CXCL-1) in A549 cells transfected with control or Rad51 siRNA at the indicated time. D Analysis of cell division on bleomycin-treated A549 cells with control or Rad51 vectors transfected by performing the EdU incorporation assay. Significance markers: *p < 0.05 compared to control; n = 3. [file 10020_2024_821_MOESM1_ESM.zip › Additional file 1/Figure S1.png]

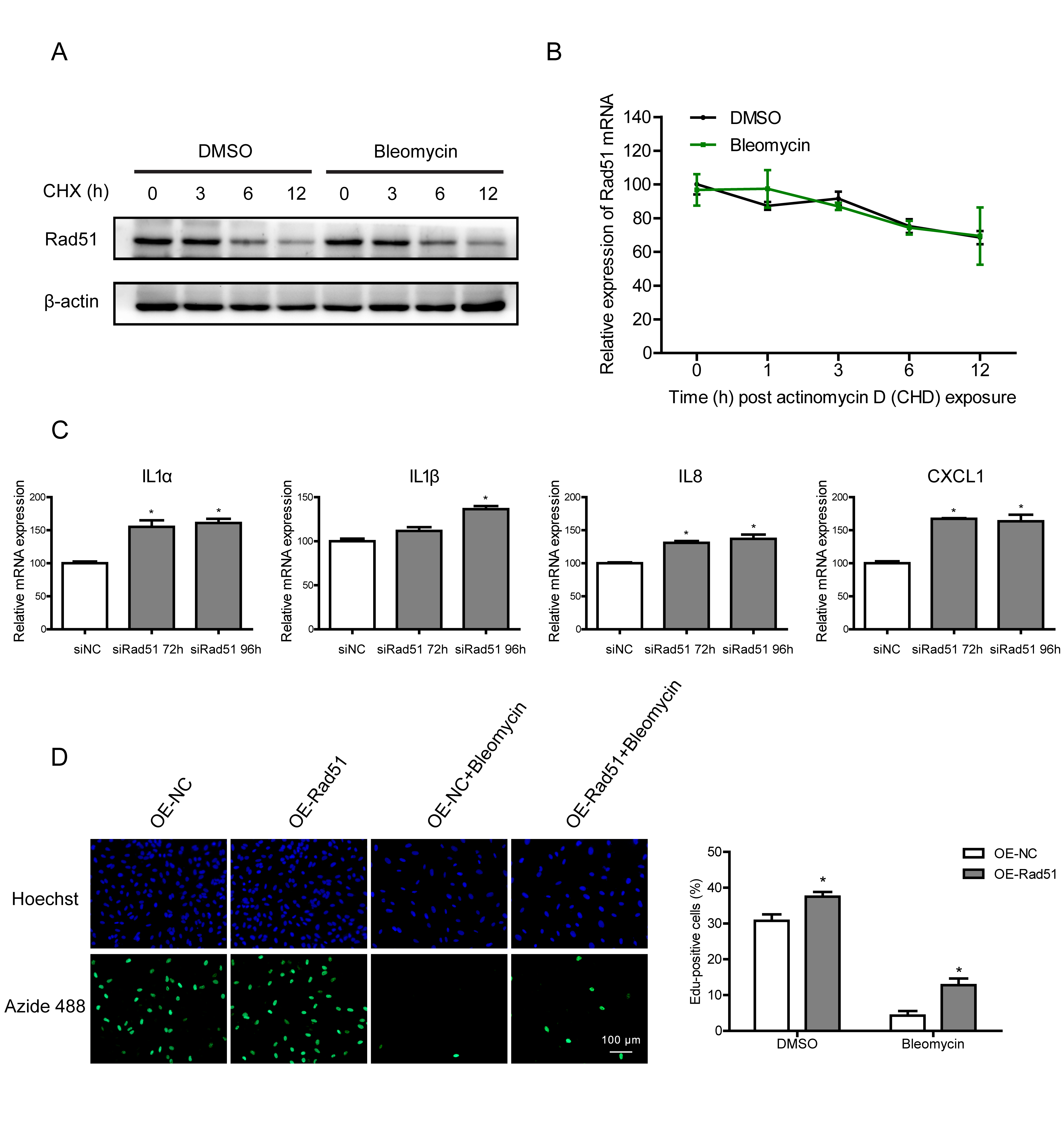

Supplement: Supplementary file 1 — Additional file 1: Figure S1. Analysis of bleomycin-induced cellular senescence and pulmonary fibrosis. A A549 cells were incubated with bleomycin at the indicated time (0, 24, 48, and 72 h), followed by SA-β-gal staining (magnification ×100). B Representative HE (Upper panel), Masson staining (Middle panel), and SA-β-Gal staining (Lower panel) of harvested mouse lung tissues. Fibrosis was evaluated using the Ashcroft score. Significance markers: *p < 0.05; **p < 0.01; ***p < 0.001 compared to control; n = 6. Figure S2. Rad51 protein and RNA stability assay, RT-PCR analysis, and EdU incorporation assay. A A549 cells were treated with 100 μg/ml CHX alone or in combination with bleomycin for 0, 3, 6, and 12 h. Then Rad51 protein expression levels were assessed by Western blotting. B A549 cells were treated with 5 μg/ml CHD alone or in combination with bleomycin for 0, 1, 3, 6, and 12 hours. The relative mRNA expression of Rad51 was assessed by RT-PCR. C RT-PCR analysis of SASP factors (IL-1α, IL-1β, IL-8, and CXCL-1) in A549 cells transfected with control or Rad51 siRNA at the indicated time. D Analysis of cell division on bleomycin-treated A549 cells with control or Rad51 vectors transfected by performing the EdU incorporation assay. Significance markers: *p < 0.05 compared to control; n = 3. [file 10020_2024_821_MOESM1_ESM.zip › Additional file 1/Figure S2.png]
